# Predictors of Treatment Adherence in Outpatients With Chronic Obstructive Pulmonary Disease: Cross-Sectional Study

**DOI:** 10.2196/83132

**Published:** 2026-09-25

**Authors:** Hien Nguyen, Trieu Nguyen, Hoai Nguyen, Giang Luong, Doanh Vu, Thong Tran

**Affiliations:** 1 Dermatology and Burn Department, Bach Mai Hospital, Hanoi, Vietnam; 2 Nam Dinh University of Nursing, Ninh Binh, Vietnam; 3 Tan Tao University, Tay Ninh Province, Vietnam; 4 Faculty of Nursing, Da Nang University of Medical Technology and Pharmacy, 99 Hung Vuong street, Hai Chau District, Da Nang, 550000, Vietnam, 0932522805; 5 Faculty of Nursing, Hai Phong University of Medicine and Pharmacy, Hai Phong, Vietnam; 6 Center for Emergency Medicine, Bach Mai Hospital, Hanoi, Vietnam; 7 Department of Emergency and Critical Care Medicine, Hanoi Medical University, Hanoi, Vietnam; 8 Faculty of Medicine, University of Medicine and Pharmacy, Vietnam National University, Hanoi, Vietnam

**Keywords:** breathing exercises, chronic obstructive pulmonary disease, COPD, inhaler use, knowledge, treatment adherence

## Abstract

**Background:**

Gaps in knowledge, inhalation skills, and adherence continue to hinder effective chronic obstructive pulmonary disease (COPD) management.

**Objective:**

The study was conducted to describe the status of COPD-related knowledge, practical skills for inhalation and breathing exercises, and treatment adherence among patients with COPD and to identify the relationships between these variables.

**Methods:**

A cross-sectional study was conducted among 420 outpatients with COPD. Data were collected in 2 rounds, including interviews to assess patients’ knowledge and treatment adherence and checklist-based observations of patients’ practical skills. Multivariable logistic regression analysis was used to evaluate the associations between knowledge and practical skills and the status of treatment adherence.

**Results:**

A total of 420 outpatients with COPD were included; 331 (78.8%) participants had inadequate COPD-related knowledge, 268 (63.8%) demonstrated adequate practical skills, and 155 (36.9%) achieved overall treatment adherence. In multivariable logistic regression, higher knowledge scores were associated with better treatment adherence (adjusted odds ratio [aOR] 1.20, 95% CI 1.06‐1.31; *P*=.001). Participants who correctly performed inhaler-use techniques were more likely to be adherent than those with incorrect technique (aOR 3.10, 95% CI 2.10‐4.70; *P*=.001). Likewise, adequate breathing-exercise skills were associated with higher adherence (aOR 1.70, 95% CI 1.08‐2.71; *P*=.02).

**Conclusions:**

Higher COPD-related knowledge and better practical skills in inhaler use and breathing exercises were associated with better treatment adherence among outpatients with COPD. Because of the cross-sectional design, these findings indicate associations rather than causal relationships. These observed associations may help inform future intervention studies evaluating patient education and skill-based training, although their effectiveness should be confirmed in longitudinal or interventional studies.

## Introduction

Chronic obstructive pulmonary disease (COPD) is becoming a major global health challenge. According to the 2024 report of the Global Initiative for Chronic Obstructive Lung Disease (GOLD), there were about 3 million deaths globally each year, and by 2060, the world is estimated to have about 5.4 million deaths related to COPD [1]. In Vietnam, the prevalence of COPD among people aged 40 years and older is 4.2%, and approximately 1.3 million people with COPD currently require diagnosis and treatment [2]. Because of the prevalence, prolonged progression, high treatment costs, and complex complications of COPD, this disease places a substantial burden on patients and health care systems worldwide and is associated with high morbidity and mortality [1].

Poor treatment adherence in COPD may be influenced by multiple factors, including inadequate disease-related knowledge, limited confidence in treatment, incorrect inhaler technique, and insufficient understanding of symptom management and breathing exercises [3]. These barriers may contribute to exacerbations, prolonged hospitalization, and poorer quality of life [1]. One of the major challenges in COPD management is ensuring that patients receive adequate education and support to understand and implement recommended self-management practices. Teaching and using an action plan in the event of worsening respiratory symptoms have been shown to reduce severity and shorten recovery time [4,5]. For patients to participate effectively in self-management programs, they must understand their disease as well as how to use medications and perform breathing exercises properly at home.

Treatment adherence is critical to the effective management of COPD and is key to addressing the growing burden of the disease [4]. For patients with COPD in the stable stage, adherence to medication and respiratory rehabilitation through the practice of breathing exercises are key to managing and alleviating symptoms, improving lung function, respiratory muscle strength, and exercise capacity, as well as reducing dyspnea and improving the quality of life [1]. However, many studies have shown that long-term adherence to chronic disease treatment is low, and it is estimated that only 50% of patients comply with treatment [6]. In addition, after the COVID-19 pandemic was brought under control in Vietnam, bed occupancy in respiratory departments of healthcare facilities has increased, coinciding with a large number of hospitalized patients with COPD exacerbations. One of the main reasons for this situation is that patients do not adhere to treatment.

To date, evidence regarding the relationship between treatment adherence, disease-related knowledge, and practical self-management skills among outpatients with COPD in Vietnam remains limited. This study aimed to assess COPD-related knowledge, practical skills in inhaler use and breathing exercises, and treatment adherence among outpatients with COPD and to examine the associations between knowledge, practical skills, and treatment adherence.

## Methods

### Study Design and Settings

A cross-sectional study was conducted among outpatients with COPD who were recruited from 3 hospitals, including Hospital C, Da Nang Hospital, and Da Nang Hospital for Lung Diseases, from April 2021 to August 2022 in Vietnam. This manuscript was prepared in accordance with the STROBE (Strengthening the Reporting of Observational Studies in Epidemiology) reporting guideline for cross-sectional studies.

### Participants and Sampling

The sample size was calculated based on the following formula:


$$n=\frac{{\left(Z_{1-\frac{\alpha }{2}}\right)}^{2}\times P\times (1-P)}{d^{2}}$$

Where Z1−α/2 is the corresponding value at the selected confidence level (equal to 1.96 if the confidence level is 95%), *P* is the treatment adherence rate (50%) [7], and *d* is the absolute error of 5%.

On the basis of the formula, the required sample size for the study was 384. In addition, the research team estimated that about 10% of participants would withdraw after signing the consent form or would not complete the survey form; therefore, the minimum sample size was set at 420. All participants were recruited using convenience sampling.

The inclusion criteria were patients diagnosed with COPD; receiving home drug therapy with inhaled medications; able to speak, read, and understand Vietnamese; and willing to participate in the study.

The exclusion criteria were patients with a history of bronchial asthma, allergic rhinitis, lung surgery, or other acute respiratory diseases; experiencing COPD exacerbations or exacerbations of comorbidities or who had experienced medication changes within the previous 3 months; or with mental disorders or other serious illnesses.

On the basis of outpatient clinic records from the 3 participating hospitals, a list of 653 patients with a physician-confirmed diagnosis of COPD who attended follow-up outpatient visits and were receiving inhaled maintenance treatment between April 2021 and August 2022 was compiled. The research team contacted these patients by telephone, explained the purpose and importance of the study, and invited them to participate. A total of 497 patients agreed to participate. Reasons for nonparticipation mainly included advanced age, poor health status, living far from the hospital, or lack of family support for transportation. After applying the inclusion and exclusion criteria at the time of data collection, 420 participants were enrolled in the final analysis.

### Measures

The questionnaire included 4 parts.

#### Part 1: Sociodemographic and Clinical Characteristics

The demographic questionnaire designed by the research team comprised 7 items (age, sex, education level, employment, years since diagnosis, smoking, and disease severity). Disease severity was assessed using the modified Medical Research Council (mMRC) questionnaire and the COPD Assessment Test (CAT). Both instruments have been translated into Vietnamese and officially published by the Ministry of Health of Vietnam and have been widely used in COPD research in Vietnam [8]. Patients were classified as having mild disease when mMRC scores were 0 to 1 and CAT scores were <10 and were classified as severe when mMRC scores were ≥2 and CAT scores were ≥10.

#### Part 2: Knowledge About COPD

Knowledge about COPD was measured using the Chronic Obstructive Pulmonary Disease Knowledge Questionnaire developed by Maples et al [9]. This scale consisted of 13 questions; each item was scored as 1 (correct) or 0 (incorrect or unknown). Overall, patients were considered to have adequate knowledge if their total score was ≥10, whereas a total score of <10 indicated inadequate disease knowledge [9]. The Vietnamese version of the scale has been evaluated in 90 people with COPD, and the results showed good content validity and internal consistency (Kuder-Richardson formula 20 was 0.883 and 0.866 before and after the test, respectively). The test-retest reliability after 4 weeks was high (*r*=0.996; *P*<.001) [10].

#### Part 3: Practical Skills Assessment

Patients’ practical skills were assessed using 2 observational checklists developed based on the 2020 Vietnamese Ministry of Health guideline for the diagnosis and treatment of COPD [11]. These assessments covered 2 domains: inhaler-use technique and breathing-exercise technique.

The inhaler-use checklist assessed patients’ ability to use metered-dose inhalers (MDIs) with 6 steps and a Turbuhaler in 6 steps. For each step, patients received 2 points for correct performance, 1 point for incorrect performance, and 0 points for failure. According to Ngo et al [8], patients were classified as having “passed” if they performed all steps correctly, and as having “failed” if at least 1 step was performed incorrectly or not performed.

The checklist for breathing exercises included pursed-lip breathing (4 steps) and diaphragmatic breathing (4 steps). For each step performed correctly, patients received 2 points, while incorrect performance received 1 point, and wrong or failed steps received 0 points. The overall assessment was classified as “passed” if the patient performed all steps of the checklist correctly and fully, and “failed” when at least one step was wrong or not performed [12].

#### Part 4: Treatment Adherence

Treatment adherence was defined as the extent to which patients followed health care providers’ recommendations regarding 2 main components of COPD treatment: inhaled medication use and breathing exercises.

Adherence to inhaled medication was assessed using the 10-item Test of Adherence to Inhalers (TAI-10), developed by Plaza et al [13]. Each item was rated on a 5-point Likert scale ranging from 1 (“worst adherence”) to 5 (“best adherence”), yielding a total score from 10 to 50. Patients with a total score of 46 to 50 were classified as adherent to inhaled medication, whereas those with a score of ≤45 were classified as nonadherent. The TAI-10 has demonstrated good reliability in previous studies, with a Cronbach α of 0.871 and a test-retest reliability coefficient of 0.832 (*P*<.01) [14].

Adherence to breathing exercises was assessed based on both the correctness of breathing-exercise performance and the maintenance of daily practice frequency according to recommendations from the GOLD and the Vietnamese Ministry of Health COPD guideline. A checklist was used to evaluate whether patients correctly performed the prescribed breathing exercises. Patients were classified as adherent to breathing exercises only if they met both of the following criteria: (1) correctly performed all required steps of the breathing exercises and (2) maintained daily practice for at least 10 to 15 minutes, with a gradual increase according to their physical capacity. Patients who performed the breathing exercises correctly but did not maintain daily practice or who practiced daily but did not perform the exercises correctly were classified as nonadherent to breathing exercises. The assessment criteria for breathing-exercise adherence were developed based on guideline recommendations and expert consensus from 5 respiratory physicians and nurses at Hospital C, Da Nang.

Overall treatment adherence was defined as adherence to both inhaled medication and breathing exercises. Patients were classified as adherent overall only if they were adherent to both components; those who were adherent to only one component or to neither component were classified as nonadherent overall. The complete set of questionnaire items is presented in Multimedia Appendix 1.

### Statistical Analysis

The study used SPSS (version 23.0; IBM Corp) for data analysis. Descriptive statistics were used to describe variables. Univariate analysis (chi-square test and independent-sample 2-tailed *t* test) was performed to examine the association between independent variables and treatment adherence. Variables with *P*<.05 (or *P*<.20 where appropriate) in univariate analysis were considered for inclusion in the multivariable model. Multivariable logistic regression analysis was then conducted to identify independent factors associated with treatment adherence, including demographic characteristics, clinical variables, knowledge, and practical skills. Adjusted odds ratios (aORs) with 95% CIs were reported. *P*<.05 was considered statistically significant.

### Ethical Considerations

The Protocol Approval Committee and the Medical Ethics Committee of Nam Dinh University of Nursing approved the study (1681/GCN-HDDD) on August 2, 2021. Participants provided written informed consent before participation.

## Results

### General Characteristics and Clinical Features of Patients With COPD

Among 420 participants, most were male (n=335, 79.8%). The mean age was 59.5 (SD 8.2) years. Most participants had a post–high school education level (n=346, 82.4%). Regarding employment status, 202 (48.1%) participants were retired, whereas only 33 (7.8%) were engaged in free trade. The mean time since diagnosis was 11.39 (SD 5.46) years, and 51.4% (n=216) of the participants were smokers (Table 1).

**Table 1.** General characteristics and clinical features of patients with chronic obstructive pulmonary disease (COPD; n=420).

Contents	Patients
Age (y), mean (SD)	59.5 (8.2)
Sex, n (%)	
Male	335 (79.8)
Female	85 (20.2)
Education level, n (%)	
High school or under	74 (17.6)
Post–high school	346 (82.4)
Employment, n (%)	
Worker and farmer	51 (12.2)
Civil servants	134 (31.9)
Retired	202 (48.1)
Free trade	33 (7.8)
Time since diagnosis (y), mean (SD)	11.39 (5.46)
Time since diagnosis (y), n (%)	
<5	84 (20)
5‐10	152 (36.2)
>10	184 (43.8)
Smoking, n (%)	
No	204 (48.6)
Yes	216 (51.4)
Disease severity, n (%)	
Mild (mMRC^a^ score 0‐1 and CAT^b^ score <10)	22 (5.2)
Severe (mMRC score ≥2 and CAT score ≥10)	398 (94.8)

a mMRC: modified Medical Research Council.

b CAT: COPD Assessment Test.

### Patients’ Knowledge About COPD

Among 420 participants, 331 (78.8%) had inadequate knowledge about COPD, whereas 89 (21.2%) had adequate knowledge. Item-level analysis showed that knowledge gaps were mainly related to smoking cessation and medication use (Table 2). Specifically, the items with the lowest correct response rates were “Quitting smoking will prevent the disease from getting worse” (n=53, 12.6%) and “Medications prevent the disease from getting worse” (n=60, 14.3%). The item with the highest correct response rate was “Shortness of breath is a symptom of COPD” (n=375, 89.2%; Multimedia Appendix 2).

**Table 2.** Patient’s knowledge, practice skills, and treatment adherence about chronic obstructive pulmonary disease (COPD; n=420).

Contents	Correct answers, n (%)
Total score of knowledge about COPD	
Adequate knowledge	89 (21.2)
Inadequate knowledge	331 (78.8)
Overall practical skills performance	
Passed	268 (63.8)
Failed	152 (36.2)
Overall treatment adherence	
Adherence	155 (36.9)
Nonadherence	265 (63.1)

### Practical Skills of Patients With COPD

Overall, of 420 participants, 152 (36.2%) failed the overall practical skills assessment, whereas 268 (63.8%) passed (Table 2). For the MDI technique, 112 (26.7%) participants failed, with the most common error being failure to exhale slowly before inhalation (77/112, 68.8%). For the Turbuhaler technique, 40 (9.5%) participants failed, and incorrect inhalation technique was the most frequent error (28/40, 70%).

Regarding breathing-exercise skills, 278 (66.2%) participants correctly performed both breathing exercises during the practical skill assessment, whereas 142 (33.8%) did not (Multimedia Appendix 3).

### Treatment Adherence of Patients With COPD

Overall treatment adherence was observed in 155 (36.9%) of 420 participants, whereas 265 (63.1%) were classified as nonadherent (Table 2). Adherence to inhaled medication was observed in 234 (55.7%) participants, and adherence to breathing exercises was observed in 265 (63.1%) participants (Multimedia Appendix 4).

### Factors Associated With Treatment Adherence of Patients With COPD

In univariate analysis, disease severity, smoking status, knowledge scores, inhaler-use skills, and breathing-exercise skills were significantly associated with treatment adherence (*P*<.05). Age, sex, education level, employment status, and duration of illness were not significantly associated with treatment adherence. Variables with *P*<.20 in univariate analysis were entered into the multivariable logistic regression model (Multimedia Appendix 5).

Knowledge scores were significantly associated with treatment adherence (adjusted odds ratio [aOR] 1.20, 95% CI 1.06‐1.31; *P*=.002). Participants who passed the inhaler-use skill assessment had higher odds of overall treatment adherence than those who failed (aOR 3.10, 95% CI 2.01‐4.75; *P*=.001). Similarly, participants who passed the breathing-exercise skill assessment had higher odds of overall treatment adherence than those who failed (aOR 1.70, 95% CI 1.08‐2.71; *P*=.02; Table 3). Among these factors, correct inhaler-use technique showed the strongest association with treatment adherence.

**Table 3.** Factors associated with treatment adherence in the multivariable logistic regression model^a^.

Factors	B	aOR^b^ (95% CI)		*P* value
Age (years)	−0.02	0.98 (0.95‐1.01)		.14
Sex				
Male	−0.41	0.66 (0.34‐1.28)		.22
Female	Reference	1.0		—^c^
Disease severity				
Mild	0.74	2.10 (0.88‐5.01)		.09
Severe	Reference	1.0		—
Smoking				
Yes	0.6	1.8 (0.85‐2.23)		.08
No	Reference	1.0		—
Knowledge score	0.17	1.2 (1.06‐1.31)		.002
Practical skills				
Inhaler-use skills				
Passed	1.2	3.1 (2.01‐4.75)		<.001
Failed	Reference	1.0		—
Breathing-exercise skills				
Passed	0.5	1.7 (1.08‐2.71)		.02
Failed	Reference	1.0		—

a Goodness-of-fit test of the logistic regression model: Hosmer-Lemeshow test, *χ*2_8_=7.6; *P*>.05.

b aOR: adjusted odds ratio.

c Not applicable.

## Discussion

### Principal Findings

This study found that COPD-related knowledge, observed practical skills, and treatment adherence among outpatients with COPD were generally suboptimal. Nearly 4 in 5 participants lacked adequate COPD-related knowledge, more than one-third demonstrated poor practical skills, and only 36.9% (155/420) achieved overall treatment adherence. In addition, better knowledge and adequate inhaler-use and breathing-exercise skills were significantly associated with higher treatment adherence.

COPD has become a major cause of morbidity and mortality worldwide. Currently, COPD cannot be completely cured, but early treatment and adherence to treatment recommendations can reduce symptoms, slow the progression of lung damage, and improve patients’ quality of life [15].

The study found that 78.8% (331/420) of patients with COPD lacked knowledge about the disease; this proportion was higher than that reported by Yang et al [16]. In addition, when comparing the correct response rates, our study and the study by Yang et al [16] showed similar patterns regarding the highest and lowest correct response rates. Specifically, the highest correct response rate was for “shortness of breath as a symptom” at 89.2% (375/420) in our study and 77.7% in the study by Yang et al [16]. This similarity can be explained by dyspnea being one of the most typical and frequent symptoms of COPD. Most people with COPD experience dyspnea when they are physically active, even in the early stages, and as the disease progresses, this problem gradually increases in intensity and severity and may occur even at rest. Two items with the lowest correct response rates were “Medications to prevent the disease from getting worse” with a correct response rate of only 14.3% (60/420) in our study compared with 10.7% in the study by Yang et al [16] and “Quitting smoking will prevent the disease from getting worse” with 12.6% (53/420) and 11.8%, respectively [16]. These knowledge assessments were related to the progression of COPD. Although most patients with COPD know that regular medication use and smoking cessation are beneficial for disease management, many do not seem to realize that with COPD, lung function cannot be fully restored [16,17]. Currently, there are no drugs or methods that can completely reverse the decline in lung function in people with COPD [17]. These findings highlight areas in which patients demonstrated substantial knowledge gaps. Educational strategies addressing these topics could be evaluated in future intervention studies.

Our study also found that 63.8% (268/420) of patients demonstrated adequate inhaler-use skills, which was higher than some studies conducted in Italy (50%) and in Korea (46.7%) but lower than that reported in a study in Pakistan (93.7%) [18-20]. Among inhaled bronchodilators used by patients, the most common errors were related to the use of the MDI and Turbuhaler, with error rates of 26.7% and 9.5%, respectively. Compared with previous studies, these rates were lower than those reported in the study in Pakistan (60% and 41%, respectively) [20] and the study by Molimard et al [21] (46.9% and 32.1%, respectively). In addition, when assessing the errors that patients often made when using the MDI and Turbuhaler, our study showed that the highest rate was for “Haven’t exhaled very slowly yet,” at 68.8% (77/112) for the MDI and 50% (20/40) for the Turbuhaler. This rate was higher than that reported by Molimard et al [21] in a study of 2935 patients with COPD hospitalized with acute exacerbations.

Most patients practiced both breathing exercises adequately (278/420, 66.2%); almost all patients passed the skills assessment when performing pursed-lip breathing (346/420, 82.4%), while diaphragm breathing exercises were still low, with 56%. This may be because diaphragmatic breathing involves more complex steps than pursed-lip breathing. These findings suggest that breathing exercises remain an important area for patient education; however, whether educational interventions improve adherence should be confirmed in future interventional studies. Additionally, our results were similar to previous research [22,23].

Treatment adherence in this study was defined as adherence to 2 key components of the prescribed COPD treatment regimen: inhaled medication use and breathing exercises. The overall treatment adherence rate was low, with only 36.9% (155/420) of participants meeting both criteria. One possible explanation is that many previous studies assessed adherence primarily in terms of medication use alone, whereas our study applied a broader and more stringent definition that required adherence to both inhaled medication and breathing exercises. This composite approach may partly explain the lower overall adherence rate observed in our sample. In addition, the study was conducted during the COVID-19 pandemic period, when limited access to health care services, reduced follow-up visits, and fear of infection may also have negatively affected treatment adherence. When inhaled medication adherence was considered separately, 55.7% (234/420) of patients were classified as adherent according to the TAI-10. This finding differs from those reported in previous studies. A study in Greece reported a high rate of nonadherence to inhaled medication (74.1%) [24], whereas studies from several European, Latin American, and Asian countries reported lower nonadherence rates [25-27]. These differences may be related to variations in study populations, health care systems, adherence assessment tools, and the operational definitions of adherence used across studies. The inclusion of breathing exercises in the definition of overall treatment adherence was based on the clinical management of COPD, which involves not only pharmacological treatment but also nonpharmacological interventions. In the present study, adherence to breathing exercises was defined not merely by correct technique but by both correct performance and maintenance of daily practice according to the prescribed recommendations. These findings suggest that inhaler technique and breathing-exercise skills may represent potential targets for future nursing interventions. However, the present cross-sectional study cannot determine whether such interventions improve treatment adherence.

In addition, higher COPD knowledge was significantly associated with greater odds of treatment adherence, suggesting that patients who better understand their disease and its management may be more likely to follow prescribed treatment recommendations. This finding is consistent with previous studies [28]. Participants who passed the inhaler-use skill assessment had 3.1-fold greater odds of overall treatment adherence than those who failed, representing the strongest association observed in the present study and consistent with previous findings [29]. Similarly, participants who passed the breathing-exercise skill assessment had 1.7 times higher odds of overall treatment adherence than those who failed, which is also in line with earlier research [16]. These findings should not be interpreted as meaning that practical skills are equivalent to adherence. Rather, they suggest that correct performance of inhaler-use techniques and breathing exercises may reflect a patient’s ability to understand, retain, and implement treatment instructions, which in turn may support better adherence behavior. In other words, practical skills in this study should be considered correlates of treatment adherence rather than direct measures of adherence itself. Because this was a cross-sectional study, the temporal direction of these associations cannot be determined, and causal relationships cannot be inferred.

Although knowledge and practical skills were significantly associated with treatment adherence in the multivariable model, these associations should be interpreted with caution. In the context of COPD management in Vietnam, adherence behavior is likely influenced not only by patients’ knowledge and self-management skills but also by broader contextual factors such as medication affordability, health insurance coverage, family support, and the quality of patient–health care provider communication. These factors may affect a patient’s ability to obtain medications, attend follow-up visits, understand treatment instructions, and maintain regular breathing exercises and may therefore be closely intertwined with both treatment adherence and the measured knowledge and skill variables. Because these contextual variables were not collected and could not be included in the regression model, residual confounding and omitted variable bias cannot be excluded. As a result, the independent contributions of COPD knowledge, inhaler-use skills, and breathing-exercise skills may have been overestimated to some extent. Therefore, the present findings should be interpreted as associations within the measured model rather than as evidence of fully independent causal effects.

### Strengths and Limitations

Treatment adherence in this study was assessed using a combination of methods, including the self-reported TAI-10 for inhaled medication adherence and a structured assessment of breathing-exercise adherence. In addition, patients’ inhaler-use and breathing-exercise skills were evaluated by direct observation using standardized checklists, which provided complementary information on practical performance.

However, this study has several limitations. First, data collection was conducted shortly after the COVID-19 pandemic was brought under control in Da Nang City, which created challenges in accessing and recruiting the target population. Although the final sample included 420 participants from 3 hospitals, caution is still needed when interpreting the representativeness of the findings. Second, treatment adherence in COPD is multidimensional and may include not only adherence to inhaled medications and breathing exercises but also smoking cessation, dietary recommendations, physical activity, and other self-management behaviors. Due to resource constraints and study feasibility, the present study focused only on adherence to inhaled medications and breathing exercises. Therefore, the findings may not fully reflect overall adherence to all recommended COPD management behaviors. Third, the study did not assess the quality of physician-patient communication or patient–health care provider relationships, which may substantially influence patients’ understanding of treatment, motivation, and adherence behaviors. Future studies should incorporate communication-related factors to provide a more comprehensive explanation of treatment adherence among patients with COPD. Fourth, some invited patients declined participation because of older age, frailty, transportation difficulties, or limited family support. In addition, patients who had experienced COPD exacerbations, exacerbations of comorbidities, or medication changes within the previous 3 months were excluded from the study. These factors may have introduced selection bias toward a relatively more stable and healthier subgroup of patients and may therefore have led to an overestimation of treatment adherence compared with the broader COPD population receiving follow-up care. Fifth, the study did not specify a minimum duration since COPD diagnosis or initiation of inhaled treatment. As a result, participants may have differed in their level of experience with disease management, inhaler use, and breathing exercises, which could have affected the homogeneity of the sample and should be considered when interpreting the findings. Finally, because this was a cross-sectional study, temporal relationships between COPD knowledge, practical skills, and treatment adherence could not be established. Therefore, causal inferences cannot be drawn. Longitudinal cohort studies and intervention trials are required to determine whether improving knowledge and practical skills leads to better treatment adherence.

### Conclusions

Higher COPD-related knowledge and better practical skills in inhaler use and breathing exercises were associated with better treatment adherence among outpatients with COPD. Because this study used a cross-sectional design, these findings demonstrate associations rather than causal relationships. The observed associations indicate that patient education and skill-based training warrant further investigation as potential nursing interventions; however, their effectiveness in improving treatment adherence should be confirmed in longitudinal cohort studies and randomized controlled trials before causal conclusions are drawn.

## Supplementary material

10.2196/83132 Multimedia Appendix 1 Responses to the Test of Adherence to Inhalers questionnaire.

10.2196/83132 Multimedia Appendix 2 Practical skills of patients with chronic obstructive pulmonary disease.

10.2196/83132 Multimedia Appendix 3 Treatment adherence of patients with chronic obstructive pulmonary disease.

10.2196/83132 Multimedia Appendix 4 The level of treatment adherence based on the clinical characteristics of patients with chronic obstructive pulmonary disease.

10.2196/83132 Multimedia Appendix 5 Study questionnaire and assessment tools used for patients with chronic obstructive pulmonary disease (COPD), including COPD knowledge questionnaire, inhaler-use technique checklists, breathing-exercise practice checklists, and adherence to inhaled medications assessed using the Test of Adherence to Inhalers.

## Abbreviations

aOR: adjusted odds ratio
CAT: COPD Assessment Test
COPD: chronic obstructive pulmonary disease
GOLD: Global Initiative for Chronic Obstructive Lung Disease
MDI: metered-dose inhaler
mMRC: modified Medical Research Council
STROBE: Strengthening the Reporting of Observational Studies in Epidemiology
TAI-10: 10-item Test of Adherence to Inhalers

## References

[1] (2024). Global strategy for prevention, diagnosis and management of COPD: 2024 report. https://goldcopd.org/2024-gold-report/.

[2] GBD 2023 Asia Chronic Respiratory Disease Collaborators (2026). Burden of chronic respiratory disease in Asia, 1990-2023: a systematic analysis for the Global Burden of Disease Study 2023. Lancet Respir Med.

[3] Świątoniowska N, Chabowski M, Polański J, Mazur G, Jankowska-Polańska B (2020). Adherence to therapy in chronic obstructive pulmonary disease: a systematic review. Adv Exp Med Biol.

[4] Alharbi MG, Kalra HS, Suri M (2021). Pulmonary rehabilitation in management of chronic obstructive pulmonary disease. Cureus.

[5] Bischof AY, Cordier J, Vogel J, Geissler A (2024). Medication adherence halves COPD patients’ hospitalization risk - evidence from Swiss health insurance data. NPJ Prim Care Respir Med.

[6] Dunbar-Jacob J, Zhao J (2025). Medication adherence measurement in chronic diseases: a state-of-the-art review of the literature. Nurs Rep.

[7] (2003). Adherence to long-term therapies: evidence for action. World Health Organization.

[8] Ngo CQ, Phan DM, Vu GV (2019). Inhaler technique and adherence to inhaled medications among patients with acute exacerbation of chronic obstructive pulmonary disease in Vietnam. Int J Environ Res Public Health.

[9] Maples P, Franks A, Ray S, Stevens AB, Wallace LS (2010). Development and validation of a low-literacy Chronic Obstructive Pulmonary Disease knowledge Questionnaire (COPD-Q). Patient Educ Couns.

[10] Nguyen TT, Upvall M, Nguyen HT, Nguyen HT, Tran LV, Nguyen PT (2023). Validity and reliability of the Vietnamese version of Chronic Obstructive Pulmonary Disease-Questionnaire for measuring knowledge of chronic obstructive pulmonary disease patients. J Health Sci Med Res.

[11] (2020). Ministry of Health's 2020 guidelines for the diagnosis and treatment of asthma (part 1) [Article in Vietnamese]. Bệnh hen.

[12] Thao LT (2022). The survey on adherence rate of breathing exercises and relationship with quality of life in patients with chronic obstructive pulmonary disease. Int J Collab Res Intern Med Public Health.

[13] Plaza V, Fernández-Rodríguez C, Melero C (2016). Validation of the “Test of the Adherence to Inhalers” (TAI) for asthma and COPD patients. J Aerosol Med Pulm Drug Deliv.

[14] Muneswarao J, Hassali MA, Ibrahim B (2021). Translation and validation of the Test of Adherence to Inhalers (TAI) questionnaire among adult patients with asthma in Malaysia. J Asthma.

[15] Liu W, Liu Y, Li X (2021). Impact of exercise capacity upon respiratory functions, perception of dyspnea, and quality of life in patients with chronic obstructive pulmonary disease. Int J Chron Obstruct Pulmon Dis.

[16] Yang H, Wang H, Du L, Wang Y, Wang X, Zhang R (2019). Disease knowledge and self-management behavior of COPD patients in China. Medicine (Baltimore).

[17] Andreas S, Hering T, Mühlig S, Nowak D, Raupach T, Worth H (2009). Smoking cessation in chronic obstructive pulmonary disease: an effective medical intervention. Dtsch Arztebl Int.

[18] Vitacca M, Paneroni M, Fracassi M (2023). Inhaler technique knowledge and skills before and after an educational program in obstructive respiratory disease patients: a real-life pilot study. Pulmonology.

[19] Jang JG, Ahn JH, Jin HJ (2021). Incidence and prognostic factors of respiratory viral infections in severe acute exacerbation of chronic obstructive pulmonary disease. Int J Chron Obstruct Pulmon Dis.

[20] Sanaullah T, Khan S, Masoom A, Mandokhail ZK, Sadiqa A, Malik MI (2020). Inhaler use technique in chronic obstructive pulmonary disease patients: errors, practices and barriers. Cureus.

[21] Molimard M, Raherison C, Lignot S (2017). Chronic obstructive pulmonary disease exacerbation and inhaler device handling: real-life assessment of 2935 patients. Eur Respir J.

[22] Sørensen D, Svenningsen H (2018). Adherence to home-based inspiratory muscle training in individuals with chronic obstructive pulmonary disease. Appl Nurs Res.

[23] Le TT, Nguyen TK, Chu TG (2023). Evaluation of the practice breathing exercises of patients with chronic obstructive pulmonary disease. Cantho J Med Pharm.

[24] Moradkhani B, Mollazadeh S, Niloofar P, Bashiri A, Oghazian MB (2021). Association between medication adherence and health-related quality of life in patients with chronic obstructive pulmonary disease. J Pharm Health Care Sci.

[25] Schreiber J, Sonnenburg T, Luecke E (2020). Inhaler devices in asthma and COPD patients - a prospective cross-sectional study on inhaler preferences and error rates. BMC Pulm Med.

[26] Şanlıtürk D, Ayaz-Alkaya S (2021). The effect of a theory of planned behavior education program on asthma control and medication adherence: a randomized controlled trial. J Allergy Clin Immunol Pract.

[27] Bissell P, May CR, Noyce PR (2004). From compliance to concordance: barriers to accomplishing a re-framed model of health care interactions. Soc Sci Med.

[28] Janjua S, Pike KC, Carr R, Coles A, Fortescue R, Batavia M (2021). Interventions to improve adherence to pharmacological therapy for chronic obstructive pulmonary disease (COPD). Cochrane Database Syst Rev.

[29] Elander A, Gustafsson M (2020). Inhaler technique and self-reported adherence to medications among hospitalised people with asthma and COPD. Drugs Real World Outcomes.

