# Supplementary material for: Predictors of Treatment Adherence in Outpatients With Chronic Obstructive Pulmonary Disease: Cross-Sectional Study
Source: JMIR Nurs. 2026 Sep 25;9:e83132. doi: 10.2196/83132 (PMC13614212; doi:10.2196/83132)
Supplement: Supplementary file 1 [file nursing-v9-e83132-s001.docx]

**Appendix Table 1. Patient’s knowledge following TAI questionnaire**

| **No** | **Contents** | **Correct answers** | **Correct answer rate (%)** |
| --- | --- | --- | --- |
| 1 | Pneumonia vaccination should be given. | Yes | 82.4 |
| 2 | Using oxygen at home can help you live longer | Yes | 82.4 |
| 3 | Medications to prevent the disease from getting worse | No | 14.3 |
| 4 | Diseases are preventable | Yes | 73.8 |
| 5 | Long acting (inhaled) bronchodilators may be stopped when symptoms improve. | No | 21.9 |
| 6 | Cough is a common symptom and won't go away | Yes | 71.0 |
| 7 | Quitting smoking will prevent the disease from getting worse | No | 12.6 |
| 8 | Smoking or secondhand smoke is the main cause | Yes | 69.3 |
| 9 | Shortness of breath as a symptom | Yes | 89.2 |
| 10 | Albuterol (inhaled form) can be used at any time when the patient finds it difficult to breathe. | Yes | 62.6 |
| 11 | Get a flu shot every year. | Yes | 79.3 |
| 12 | Inhalation should only be used when breathing is difficult | No | 21.2 |
| 13 | Can be cured | No | 61.9 |
