# Supplementary material for: Predictors of Treatment Adherence in Outpatients With Chronic Obstructive Pulmonary Disease: Cross-Sectional Study
Source: JMIR Nurs. 2026 Sep 25;9:e83132. doi: 10.2196/83132 (PMC13614212; doi:10.2196/83132)
Supplement: Supplementary file 2 [file nursing-v9-e83132-s002.docx]

**Appendix Table 2. Practical skills of patients with COPD**

| **Cotents** | | **N (%)** |
| --- | --- | --- |
| **I. Practice with using inhaled drugs** | | |
| **Practice with Metered dose inhaler** | **Total failed** | **112 (26.7)** |
|  | Haven't exhaled very slowly yet | 77 (68.8) |
|  | The mouth of the tool has not been covered | 57 (50.9) |
|  | Push and inhale error | 38 (33.9) |
|  | Haven't breathed for 10 seconds | 57 (50.9) |
| **Practice with Turbuhaler** | **Total failed** | **40 (9.5)** |
|  | Haven't exhaled very slowly yet | 20 (50.0) |
|  | Inhalation is not correct | 28 (70.0) |
|  | Haven't held breath for 10 seconds | 26 (65.0) |
| **II. Practice with breathing exercises** | | |
| **Breathing exercises** | **Practice pursed-lip breathing** | |
|  | Passed | 348 (82.4) |
|  | Failed | 74 (17.8) |
|  | **Diaphragm breathing** | |
|  | Passed | 235 (56) |
|  | Failed | 185 (44) |
|  | **Combine 2 type of breathing exercises** | |
|  | Passed | 278 (66.2) |
|  | Failed | 142 (33.8) |
