# Supplementary material for: Predictors of Treatment Adherence in Outpatients With Chronic Obstructive Pulmonary Disease: Cross-Sectional Study
Source: JMIR Nurs. 2026 Sep 25;9:e83132. doi: 10.2196/83132 (PMC13614212; doi:10.2196/83132)
Supplement: Supplementary file 3 [file nursing-v9-e83132-s003.docx]

**Appendix Table 3. Treatment adherence of patients with COPD**

| Contents | N, % |
| --- | --- |
| Adherence to inhaled drugs (by TAI-10) | |
| Adherence | 234 (55.7) |
| Non-adherence | 186 (44.3) |
| Adherence to breathing exercises | |
| Adherence | 265 (63.1) |
| Non-adherence | 155 (36.9) |
