# Supplementary material for: Predictors of Treatment Adherence in Outpatients With Chronic Obstructive Pulmonary Disease: Cross-Sectional Study
Source: JMIR Nurs. 2026 Sep 25;9:e83132. doi: 10.2196/83132 (PMC13614212; doi:10.2196/83132)
Supplement: Supplementary file 4 [file nursing-v9-e83132-s004.docx]

**Table 4. The level of treatment adherence based on the clinical characteristics of patients with COPD**

| **Clinical characteristics** | **Adherence** | **Non-adherence** | **OR (95%Cl)** | ***P-value*** |
| --- | --- | --- | --- | --- |
| **Age (Mean±SD)** | 34.7 ± 5.2 | 28.9 ± 6.1 | 1.11  (0.9 – 1.3) | .11 |
| **Gender** | | | | |
| Male | 130 (40.1) | 194 (59.9) | 0.53  (0.3 – 0.9) | .12 |
| Female | 71 (74.0) | 25 (26.0) |  |  |
| **Education level** | | | | |
| High school or under | 27 (43.5) | 35(56.5) |  | .24 |
| Post-high school | 128 (35.8) | 230 (64.2) |  |  |
| **Employment** | | | | |
| Worker and Famer | 16 (34.8) | 30 (65.2) | - | .87 |
| Civil servants | 38 (40.4) | 56 (59.6) |  |  |
| Retired | 87 (35.8) | 156 (64.2) |  |  |
| Free trade | 14 (37.8) | 23 (62.2) |  |  |
| **Year of illness** | | | | |
| <5 years | 25 (39.7) | 38 (60.3) | - | .99 |
| 5-10 years | 56 (31.1) | 124 (68.9) |  |  |
| >10 years | 74 (41.8) | 103 (58.2) |  |  |
| **Disease severity** | | | | |
| Mild | 15 (68.2) | 7 (31.8) | 0.3  (0.1 – 0.6) | .002 |
| Severe | 140 (35.2) | 258 (64.8) |  |  |
| Smoking |  |  |  |  |
| Yes | 65 (31,9) | 139 (68,1) | 1,53  (1.0 – 2.3) | .037 |
| No | 90 (41,7) | 126 (58,3) |  |  |
| **Patient’s knowledge (Mean±SD**) | 7.88 ± 2.07 | 8.89 ± 2.27 | 1.2  (1.06 –1.31) | .001 |
| **Practice with using inhaled drugs** | | | | |
| Passed | 82 (53.9) | 70 (46.1) | 3.1  (2.1 – 4.7) | .001 |
| Failed | 73 (27.2) | 195 (72.8) |  |  |
| **Practice with breathing exercises** | | | | |
| Passed | 112 (40.3) | 116 (59.7) | 1.6  (1.1 – 2.4) | .04 |
| Failed | 43 (30.3) | 99 (69.7) |  |  |
