# Supplementary material for: Predictors of Treatment Adherence in Outpatients With Chronic Obstructive Pulmonary Disease: Cross-Sectional Study
Source: JMIR Nurs. 2026 Sep 25;9:e83132. doi: 10.2196/83132 (PMC13614212; doi:10.2196/83132)
Supplement: Supplementary file 5 [file nursing-v9-e83132-s005.docx]

**Part 1: KNOWLEDGE ABOUT CHRONIC OBSTRUCTIVE PULMONARY DISEASE (COPD-Q)**

| **No.** | **Items** | **Correct** | **Incorrect** | **Don’t know** |
| --- | --- | --- | --- | --- |
| 1 | People with COPD should get vaccinated against pneumonia. |  |  |  |
| 2 | Using oxygen at home can help you live longer |  |  |  |
| 3 | COPD medications prevent the disease from getting worse. |  |  |  |
| 4 | COPD can be prevented |  |  |  |
| 5 | Patients can stop long-acting bronchodilators (inhalers) when their symptoms get better. |  |  |  |
| 6 | Coughing is a common symptom of COPD and will not go away |  |  |  |
| 7 | Stopping smoking will prevent COPD from getting worse |  |  |  |
| 8 | Smoking or secondhand smoke is a major cause of COPD |  |  |  |
| 9 | COPD patients may feel short of breath |  |  |  |
| 10 | Albuterol (inhaler) can be used anytime you feel short of breath. |  |  |  |
| 11 | COPD patients should get a flu shot every year. |  |  |  |
| 12 | Patients should only use inhaled COPD medications when they are short of breath |  |  |  |
| 13 | COPD can be cured |  |  |  |

**Part 2: PRACTICE USING INHALED MEDICINES**

**Metered dose inhaler usage procedure checklist (MDI)**

| **Steps** | **Not do** | **Done but not correct** | **Done correct** |
| --- | --- | --- | --- |
| Step 1: Open the medicine bottle |  |  |  |
| Step 2: Shake the medicine bottle well |  |  |  |
| Step 3: Exhale slowly as much as possible |  |  |  |
| Step 4: Close the mouthpiece |  |  |  |
| Step 5: Press the tip of the medicine bottle and inhale deeply |  |  |  |
| Step 6: Hold breath for at least 10 seconds. |  |  |  |

Remember to rinse your mouth after inhaling the drug.

**Turbuhaler dry powder inhaler usage checklist**

| **Steps** | **Not do** | **Done but not correct** | **Done correct** |
| --- | --- | --- | --- |
| Step 1: Unscrew and remove the cap of the inhaler |  |  |  |
| Step 2: Hold the inhaler upright, load the medication by turning the base all the way to the right and then turning it back to the original position until you hear a click to indicate the medication is loaded |  |  |  |
| Step 3: Exhale fully before closing the inhaler |  |  |  |
| Step 4: Close the inhaler and inhale deeply until you can no longer inhale |  |  |  |
| Step 5: Hold your breath for 10 seconds |  |  |  |
| Step 6: Replace the cap of the inhaler |  |  |  |

Remember to rinse your mouth after inhaling the drug.

**Part 3: PRACTICE THERAPY OF BREATHING EXERCISES**

**Pursed lip breathing technique checklist**

| **Steps** | **Not do** | **Done but not correct** | **Done correct** |
| --- | --- | --- | --- |
| Step 1: Sit comfortably |  |  |  |
| Step 2: Relax your neck and shoulders. |  |  |  |
| Step 3: Inhale slowly through your nose. |  |  |  |
| Step 4: Purse your lips as if you are whistling, exhale slowly through your mouth so that the exhalation time is twice as long as the inhalation time. |  |  |  |

**Diaphragmatic breathing technique checklist**

| **Steps** | **Not do** | **Done but not correct** | **Done correct** |
| --- | --- | --- | --- |
| Step 1: Sit or lie down comfortably and place your hands on your belly. |  |  |  |
| Step 2: Relax the muscles in your neck and shoulders. Place one hand on your belly and the other on your chest. |  |  |  |
| Step 3: Inhale slowly through your nose so that the hand on your belly feels like your belly is expanding. Your rib cage should not move. |  |  |  |
| Step 4: Pull your belly in and exhale slowly through your mouth, exhaling for twice as long as you inhale, and feel your belly sinking in with the hand on your belly. |  |  |  |

**Part 4: COMPLIANCE WITH INHALATION MEDICATIONS** (Test of the adherence to inhalers TAI)

| **No.** | **Items** | **Scale** | | | | |
| --- | --- | --- | --- | --- | --- | --- |
|  |  | **Alway (1)** | **Usually (2)** | **Sometimes (3)** | **Seldom (4)** | **Never**  **(5)** |
| 1 | During the past 7 days, how many times have you forgotten to take your regular inhaler? |  |  |  |  |  |
| 2 | Have you forgotten to take your inhaler? |  |  |  |  |  |
| 3 | Have you stopped taking your inhaler when you feel better? |  |  |  |  |  |
| 4 | Have you stopped taking your inhaler when you are on vacation or on weekends? |  |  |  |  |  |
| 5 | Have you stopped taking your inhaler when you are anxious or sad? |  |  |  |  |  |
| 6 | Have you stopped taking your inhaler because you are afraid of side effects? |  |  |  |  |  |
| 7 | Have you stopped taking your inhaler because you believe it is not treating your condition? |  |  |  |  |  |
| 8 | Have you inhaled less than your doctor prescribed? |  |  |  |  |  |
| 9 | Have you stopped taking your inhaler because you believe it is interfering with your daily life or work |  |  |  |  |  |
| 10 | Have you stopped taking your inhaler because you have difficulty paying for it? |  |  |  |  |  |
| 11 | Does the patient remember the prescribed regimen (dose and frequency)? (check medical records) | Yes | | | No | |
| 12 | Patient's inhaler technique is rated as * (check inhaler technique) | Serious mistake | | | No serious errors | |
